# Glucose ingestion increases passive absorption of a nutrient‐sized solute, mannitol, in healthy young adults

**DOI:** 10.14814/phy2.70427

**Published:** 2025-06-16

**Authors:** G. Patrick Lambert, Caroline Jachino, Liam Murphy, Katherine Krueger

**Affiliations:** ^1^ Department of Exercise Science and Pre‐Health Professions Creighton University Omaha Nebraska USA

**Keywords:** absorption, glucose, intestine, mannitol, paracellular

## Abstract

Active glucose absorption increases passive, paracellular absorption of small solutes. Absorption of larger molecules by this mechanism has not been verified in humans under physiological conditions. The purpose of this study was to determine if ingestion of a glucose solution enhances absorption of mannitol in humans. Mannitol (182 Da) is a non‐metabolizable molecule believed to be absorbed only via the paracellular route. Its urinary excretion therefore may serve as an index for paracellular absorption of similar‐sized solutes, such as glucose (180 Da) and amino acids (average 138 Da). Eight healthy individuals (five females, three males; mean age = 22 +/− 1 yrs) ingested a 4% glucose/0.2% mannitol solution or a 4% fructose/0.2% mannitol solution using a randomized, balanced design. Urine was collected for 5 h and mannitol excretion determined. Ingestion of the glucose solution increased (*p* < 0.05) mannitol excretion (0.52 +/−0.27 g) compared to the fructose solution (0.39 +/−0.13 g), a 33% increase. These results indicate glucose promotes passive absorption of nutrient‐sized solutes, likely via the paracellular route. This may explain how humans can absorb high amounts of glucose when maximal active transport is exceeded. Furthermore, other nutrients such as amino acids may utilize this route, thereby enhancing absorption.

## 1 INTRODUCTION

The ability of the small intestine to efficiently absorb large amounts of carbohydrate when luminal glucose concentration exceeds the capacity of the sodium‐glucose cotransporter (SGLT1) is likely due to enhanced passive transport. The passive mechanism may be due to the “transient insertion” of GLUT2 into the apical membrane of enterocytes (Kellett & Helliwell, 2000) or via the paracellular pathway following the opening of tight junctions upon activation of SGLT1 (Madara, 1994; Madara & Pappenheimer, 1987; Pappenheimer & Reiss, 1987). Its role in overall glucose absorption has also been debated as some have found it to be insignificant (Fine et al., 1993) while others have found it to be a major route when luminal glucose concentrations are high (Turner et al., 2000). The discrepancies are likely due to methodological differences regarding water availability to promote solvent drag through paracellular spaces (Madara, 1994). Paracellular glucose absorption via solvent drag would explain the ability to absorb large amounts of glucose during the ingestion of oral rehydration solutions and “sport drinks” in humans. Under such conditions, glucose concentration in the small intestine can easily exceed 100 mM, more than 3 times the active transport capacity of SGLT1 (~30 mM) (Gromova et al., 2021). Furthermore, high rates of glucose transport are accompanied by high rates of water absorption with the ingestion of such solutions (Gisolfi et al., 1998). However, the largest reported molecule to be transported paracellularly following the ingestion of a glucose solution is creatinine (113 Da) (Turner et al., 2000). Given that glucose (180 Da) is larger than this, it is unclear whether it can be absorbed paracellularly under normal physiological conditions.

Based on this, the intent of the present investigation was to determine if ingestion of a glucose solution enhances paracellular absorption of a glucose‐sized molecule in young, healthy humans. Mannitol (182 Da) is a hydrophilic, non‐metabolizable molecule that is believed to be transported only paracellularly (Naftalin, 2014; Ordiz et al., 2018). Thus, it can serve as a probe for passive, paracellular absorption of hydrophilic molecules of its size or smaller (e.g., glucose). It was hypothesized that mannitol absorption would be significantly greater following ingestion of a glucose‐containing solution (via activation SGLT1), compared to a fructose‐containing solution (which is absorbed by facilitated diffusion through GLUT5), due to SGLT1‐elicited opening of tight junctions and subsequent paracellular absorption by solvent drag (Pappenheimer & Reiss, 1987; Turner et al., 2000). The findings should provide further knowledge as to whether glucose, and possibly other nutrients, are significantly absorbed via the paracellular route.

## 2 MATERIALS AND METHODS

Eight healthy individuals (five females, three males; mean age = 22 +/− 1 yrs) participated after providing written informed consent. Subjects were screened with a health history questionnaire for existing health conditions including GI symptoms/disorders and were instructed not to ingest nonsteroidal anti‐inflammatory drugs (NSAIDs), alcohol, or perform intense exercise for at least 48 h prior to being tested, as each of these is known to affect intestinal permeability. Subjects consumed the same diet the 24 h prior to each experiment verified by food logs. All procedures were approved by the Creighton University Institutional Review Board and the study was conducted in accordance with the Declaration of Helsinki.

Two experiments (based on a randomized, balanced design) were conducted on each subject to test the effects of glucose or fructose on passive intestinal absorption of mannitol. Experiments consisted of ingestion of 1000 mL of either a glucose/mannitol solution or a fructose/mannitol solution. Each solution contained either 4% glucose or 4% fructose (i.e., 40 g/L; 222 mM) and 0.2% mannitol (2 g/L; 11 mM). Solutions were flavored with one packet of Gatorade Zero® and the flavor was the same for each experiment. Each packet contained the following ingredients: citric acid, sodium citrate, salt, monopotassium phosphate, sucralose, natural flavor, silica dioxide, acesulfame potassium, yellow 6, and tocopherols (flavor protectant). The sodium and potassium content per packet was 230 mg and 70 mg, respectively, resulting in a sodium concentration of 10 mEq/L and a potassium concentration of 1.8 mEq/L. Calculated solution osmolality was 257 mOsm/kg H_2_O based on the concentrations of glucose, fructose, mannitol, sodium, and potassium (and any associated anions). Measured osmolality averaged 265 mOsm/kg H_2_O reflecting the additional ingredients in the flavoring.

Solutions were ingested over the span of 2 h (200 mL every 30 min) and subjects were asked not to eat or drink anything except water, if necessary, during that period and for the following 3 h. All urine produced during the total 5‐h experiment (with time zero at the beginning of ingestion) was collected in a 3‐L urine collection container. The container was kept cold in a cooler with ice packs to inhibit bacterial growth. At the end of the experiment, subjects voided their bladder one final time into the container. Total 5‐h urine volume and urine specific gravity were measured and recorded, and aliquots were collected and frozen at −20°C for subsequent analysis of mannitol.

Mannitol concentration was determined using a commercially available assay kit (catalog number EMNT‐100; BioAssay Systems, Hayward, CA). Urinary excretion of ingested mannitol was determined by multiplying the urine mannitol concentration by the total urine volume. Urine specific gravity was determined by hand refractometry to assess hydration status. Solution osmolality was measured by freezing‐point depression (Multi‐Osmette Osmometer, Precision Systems, Natick, MA).

A power test based on previous data (Turner et al., 2000) was conducted and indicated eight subjects would provide >80% power to detect significant differences. The Shapiro–Wilk test was employed to test for normality of the data, and a Student *t*‐test was utilized to test for significant differences between the two conditions. The level of significance was set at *p* < 0.05.

## 3 RESULTS

**FIGURE 1:**
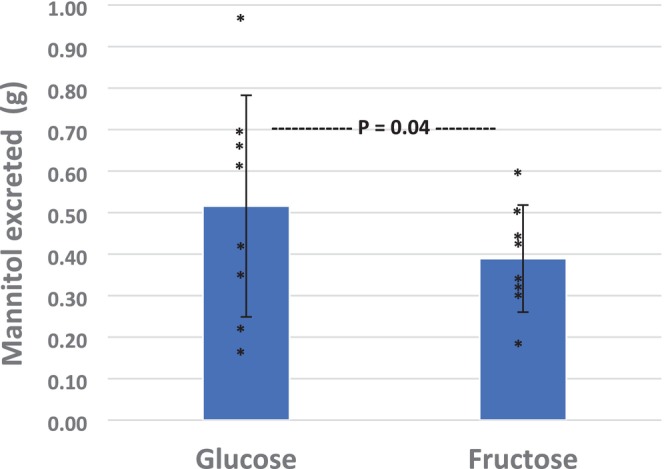
Urinary mannitol excretion in the two experimental conditions. The glucose condition was significantly greater (*p* = 0.04) than the fructose condition. Both mean (bar graph; +/− SD) and individual data points are presented.

There was no significant difference in total urine volume produced (841 +/− 356 mL vs. 918 +/− 391 mL) or urine specific gravity (1.005 +/− 0.002 vs. 1.005 +/− 0.002) for the glucose and fructose experiments, respectively (Table 1). However, ingestion of the glucose‐containing solution significantly (*p* = 0.04) increased urinary mannitol excretion (0.52 +/− 0.27 g) compared to ingestion of the fructose‐containing solution (0.39 +/− 0.13 g) (Table 2; Figure 1).

**TABLE 1:** Total urine volume and urine specific gravity for the two experimental conditions.

Experiment	Total urine volume (mL)	Urine specific gravity
Glucose condition	841 ± 356	1.005 ± 0.002
Fructose condition	918 ± 391	1.005 ± 0.002

*Note*: Values are means +/− S.

**TABLE 2:** Individual data for total urinary mannitol excretion in each experiment.

Subject	Glucose condition (g)	Fructose condition (g)
1	0.96	0.60
2	0.70	0.51
3	0.62	0.44
4	0.42	0.32
5	0.67	0.43
6	0.23	0.31
7	0.36	0.33
8	0.17	0.19
Mean (+/− SD)	0.52 (0.27)*	0.39 (0.13)

* Significantly (*p* = 0.04) greater than fructose condition.

## 4 DISCUSSION

The results of the present study indicate that ingestion of a glucose‐containing solution significantly increases absorption of mannitol, which is a hydrophilic solute with a similar molecular weight as glucose and is believed to be absorbed passively and paracellularly. These results support those of Turner et al. (2000) who found that ingestion of a glucose solution increased absorption of a smaller hydrophilic solute, creatinine, also believed to be absorbed paracellularly, and extend those results to larger molecules up to the size of monosaccharides and amino acids. It should be noted that mannitol may not be a totally accurate paracellular probe for such molecules, as its stereochemistry in solution as a sugar alcohol may be different than that of glucose (a closed pyranose ring) or amino acids (charged). The results, however, do suggest that passive paracellular transport may be an important mechanism for glucose absorption when glucose concentrations are high in the intestinal lumen. An example of this would be when humans ingest carbohydrate‐electrolyte “sport drinks” or oral rehydration solutions in which the glucose concentration in the proximal small intestine can easily exceed 100 mM (Gisolfi et al., 1998), a concentration 2–4 times higher than that which saturates SGLT1 (i.e., 30–50 mM) (Kellett & Helliwell, 2000; Yang et al., 2011). Since symptoms such as gas, bloating, cramps, and/or diarrhea do not normally occur when ingesting large volumes of such solutions, it is unlikely a significant amount of unabsorbed glucose is reaching the colon, resulting in bacterial degradation. Thus, it appears the glucose must be getting absorbed in the small intestine by another route. Our results indicate this includes the paracellular pathway since our probe for glucose absorption (i.e., mannitol) is not absorbed by glucose transporters (Naftalin, 2014; Ordiz et al., 2018) and no other passive pathways have been identified for mannitol. Therefore, both GLUT2 (Kellett & Helliwell, 2000) and paracellular transport likely allow for efficient glucose uptake when glucose concentrations are high. Our data indicate that the presence of glucose increased paracellular absorption by 33% compared to when glucose was not present (i.e., the fructose condition in which SGLT1 was not utilized; fructose is absorbed by facilitated diffusion via GLUT5). In support of this finding, Shi and Gisolfi (1996) found that “blocking” the paracellular route with protamine reduced glucose absorption 50% in the rat during intestinal perfusion with a solution containing 150 mM glucose (Shi & Gisolfi, 1996). Finally, numerous studies examining absorption of carbohydrate‐electrolyte solutions have shown high rates of carbohydrate (e.g., glucose and fructose) absorption occurring simultaneously with rapid water absorption in the proximal small intestine (Gisolfi et al., 2001; Lambert et al., 1996, 1997, 2008, 2011; Rogers et al., 2005; Shi et al., 1994). In contrast, Fine et al. (1993), using the intestinal perfusion technique, did not observe a significant stimulatory effect of glucose on paracellular absorption of permeability probes such as mannitol. The results of that study have been challenged, however, (Madara, 1994) as it is likely insufficient water transport occurred to allow for paracellular absorption via solvent drag. As a matter of fact, a follow‐up study by the same authors found that intestinal infusion of glucose did enhance passive absorption of L‐xylose (150 Da) under conditions of concurrent water absorption (Fine et al., 1994).

The current results enhance our understanding of how the small intestine can efficiently absorb high glucose loads in the small intestine during ingestion of large amounts of carbohydrate‐electrolyte drinks and how recovery beverages containing water, carbohydrate, electrolytes, and protein could promote simultaneous rehydration, glycogen resynthesis, and protein synthesis during recovery from exercise (Ferguson‐Stegall et al., 2011; Ivy et al., 2002; James et al., 2013). Further studies in this area should be conducted.

These results also may have implications regarding potential adverse effects on blood glucose levels with ingestion of “sugary” beverages such as soft drinks and the possible relationship this may have to conditions such as metabolic syndrome and diabetes mellitus. It has been found, for example, that diabetes mellitus results in upregulation of both SGLT1 and GLUT2 in rats and increases intestinal permeability (i.e., increased paracellular absorption) to mannitol in humans (Gromova et al., 2021). Such adaptations likely allow for greater glucose absorption but also likely increase blood glucose levels above what might occur in individuals without diabetes. Research is ongoing in this area (Gromova et al., 2021).

In conclusion, the present results indicate that ingestion of a glucose solution, at glucose concentrations above the maximal rate of transport for SGLT1, stimulates enhanced passive absorption of mannitol, a hydrophilic molecule the size of glucose and larger than most amino acids. This does not appear to be due to increased facilitated diffusion via GLUT2 since mannitol does not likely utilize that route. Therefore, the absorption pathway may be paracellular via glucose‐elicited opening of tight junctions. These results support findings from previous studies in this area in healthy humans and may further explain how high concentrations of glucose (and possibly other nutrients) can be efficiently absorbed in the human small intestine when other routes of transport are saturated. These findings should also be considered in regard to blood glucose homeostasis and the absorption of other nutrients such as water and amino acids both during and in recovery from exercise.

## AUTHOR CONTRIBUTIONS

GPL: Conceived and designed research, performed experiments, analyzed data, interpreted results of experiments, prepared figures, drafted manuscript, edited and revised manuscript, and approved final version of manuscript. CJ: Performed experiments, interpreted results of experiments, edited and revised manuscript, and approved final version of manuscript. LM: Performed experiments, interpreted results of experiments, prepared figures, edited and revised the manuscript, and approved the final version of the manuscript. KK: Performed experiments, interpreted results of experiments, edited and revised the manuscript, and approved the final version of the manuscript.

## FUNDING INFORMATION

This research was funded by a *Magis! Investigatio* Research Award from the Center for Undergraduate Research and Scholarship at Creighton University (to GPL).

## CONFLICT OF INTEREST STATEMENT

The authors declare no conflicts of interest. The funders had no role in the design of the study; in the collection, analyses, or interpretation of data; in the writing of the manuscript; or in the decision to publish the results.

## ETHICS STATEMENT

This study was approved by the Institutional Review Board at Creighton University. All subjects provided written informed consent and the study was conducted in accordance with the Declaration of Helsinki.

## Data Availability

All data supporting the conclusions from this study are found within the manuscript.
